# Prenatally Diagnosed Beare‐Stevenson Cutis Gyrata Syndrome With a Novel *FGFR2* Variant

**DOI:** 10.1002/pd.70113

**Published:** 2026-03-03

**Authors:** Haley M. Crane, Rose Giardine, Alanna Strong, K. Taylor Wild, Elaine Zackai, Lorraine Dugoff, Teresa N. Sparks, Beverly Coleman, Julie S. Moldenhauer

**Affiliations:** ^1^ Richard D. Wood Jr. Center for Fetal Diagnosis and Treatment Children's Hospital of Philadelphia Philadelphia Pennsylvania USA; ^2^ Division of Reproductive Genetics Department of Obstetrics and Gynecology Perelman School of Medicine at the University of Pennsylvania Philadelphia Pennsylvania USA; ^3^ Division of Maternal‐Fetal Medicine Department of Obstetrics and Gynecology Perelman School of Medicine at the University of Pennsylvania Philadelphia Pennsylvania USA; ^4^ Division of Human Genetics Children's Hospital of Philadelphia Philadelphia Pennsylvania USA; ^5^ Division of Neonatology Children's Hospital of Philadelphia Philadelphia Pennsylvania USA; ^6^ Center for Maternal‐Fetal Precision Medicine University of California San Francisco San Francisco California USA; ^7^ Department of Obstetrics, Gynecology, and Reproductive Sciences University of California San Francisco San Francisco California USA; ^8^ Department of Radiology Perelman School of Medicine at the University of Pennsylvania Philadelphia Pennsylvania USA; ^9^ Department of Surgery Perelman School of Medicine at the University of Pennsylvania Philadelphia Pennsylvania USA

**Keywords:** Beare‐Stevenson cutis gyrata syndrome, *FGFR2*, genome sequencing, prenatal diagnosis

## Abstract

- What is already known about this topic?
  ◦ Heterozygous *FGFR2* variants cause a spectrum of craniosynostosis disorders, including Beare‐Stevenson cutis gyrata syndrome (BSS).
  ◦ BSS has been previously reported in association with specific gain‐of‐function variants (p.Ser372Cys and p.Tyr375Cys).
- What does this study add?
  ◦ We report a prenatally diagnosed case of BSS, a disorder for which the fetal phenotype has rarely been described.
  ◦ This is the first report of a variant at this position (p.Phe276Cys) in association with BSS, thus expanding the known genotype‐phenotype correlation.

## 1 Fetal Phenotype

Forty one‐year‐old G5P2022 patient referred for hydrops fetalis (*skin edema around the fetal cranium and face extending down to the abdomen, mild ascites, thickened nuchal fold measuring up to 18 mm*) and multiple congenital anomalies. The pregnancy was conceived via IVF with PGT‐A (euploid male). Notable family history included a paternal uncle with cleft palate and another paternal uncle with imperforate anus. First trimester ultrasound identified an increased nuchal translucency (7 mm) (Table 1A).

**TABLE 1A:** Clinical data.

Case	Parental details			Gestation at diagnosis	Phenotypes (HPO terms)	Obstetric history	Family history	Outcome
1	Maternal	Age	41	25 weeks 2 days	Increased nuchal translucency (HP:0010880) Tracheoesophageal fistula (HP:0002575) Pelvic kidney (HP:0000125) Hypospadias (HP:0000047) Micropenis (HP:0000054) Hypertelorism (HP:0000316) Proptosis (HP:0000520) Low set ears (HP:0000369) Pectus excavatum (HP:0000767) Hepatomegaly (HP:0002240) Midface retrusion (HP:0011800) Macroglossia (HP:0000158) Thymus hyperplasia (HP:0010516)	G5P2022	Paternal uncle of fetus with isolated cleft palate; another paternal uncle with isolated imperforate anus	Repeat cesarean delivery at 35 weeks of male neonate (3.86 kg, > 97%). Additional postnatal phenotype included brachycephaly, skin redundancy, natal teeth, cutis gyrata, edematous ears with linear divets, large/contracted left thumb, possible contractures of the digits, lower extremity 2‐3 syndactyly, right nipple skin tag, large umbilical stump, facial hemangioma, and limited movement. Parents elected for comfort care and the infant expired on DOL 50.
		Ethnicity	Russian, Ashkenazi Jewish					
	Paternal	Age	42					
		Ethnicity	Ukrainian, Russian, Ashkenazi Jewish					

**FIGURE 1:**
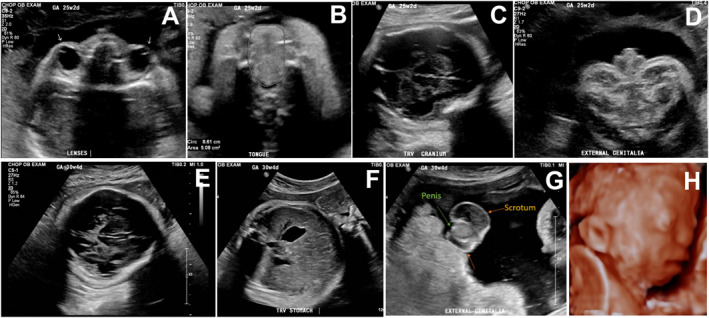
High‐resolution ultrasound imaging at the time of initial evaluation at 25 weeks 2 days gestation demonstrating concern for fetal (A) proptosis, (B) macroglossia, (C) incompletely ossified cranium, and (D) microphallus. Follow‐up high resolution ultrasound imaging performed at 30 weeks 4 days gestation demonstrating (E) abnormal fetal skull shape, (F) small stomach, (G) small shaft with unusual scrotal shape, and (H) 3D depiction of the fetal face.

Ultrasound at our center at 25 weeks 2 days gestation revealed multiple anomalies including craniofacial (*brachycephaly, focal regions of abnormal/absent calvarial ossification, midface hypoplasia, depressed nasal bridge, macroglossia, hypertelorism, proptosis, unopposed eyelids, elongated/low set ears*), skeletal (*thoracolumbar segmentation anomalies, pectus excavatum*), gastrointestinal (*hepatomegaly, possible esophageal atresia/tracheoesophageal fistula*), genitourinary (*left pelvic kidney, flattened left adrenal gland, non‐visualized perineum/anal region, hypospadias, microphallus*), and enlarged thymus. Fetal echocardiogram was normal. Ultrasound at 30 weeks 4 days identified abnormal skull shape with extremely thickened skin over the posterior neck appearing to be rugated and continued concern for abnormal genitalia (*small shaft with unusual scrotal shape*) (Figure 1A–H).

## 2 Diagnostic Method

Amniocentesis at 21 weeks 5 days revealed a normal microarray and negative cytomegalovirus/toxoplasmosis PCR. Genome sequencing (GS) was recommended following our evaluation. Proband‐only GS was performed by the Clinical Laboratory Improvement Amendments (CLIA)‐approved UCSF Genomic Medicine Laboratory using DNA isolated from cultured amniocytes. Parental samples were used for variant phasing and Sanger confirmation (Table 1B).

**TABLE 1B:** Genetic findings.

Procedure (gest age)	Direct/culture?	Performed test	Secondary confirmatory test	Gene (name; REFSEQ)	Known disease (OMIM)	Variant	ACMG classify‐cation	Criteria applied	Inheritance & zygosity	Interpret‐ation
Amniocentesis (21 weeks 5 days)	Culture	Proband‐only genome sequencing	Sanger confirmed in fetal and parental specimens	*FGFR2* (NM_000141.5)	Beare‐Stevenson cutis gyrata syndrome (MIM# 123790)	Allele 1: c.827 T > G (p.Phe276Cys)	Likely pathogenic	PS2 PM2 PM5 PP3	De novo, heterozygous	Causative

Sequencing via NGS technology was performed using the NovaSeq 6000 (Illumina) sequencer and the KAPA EvoPlus library prep kit (Roche). Alignment and variant calling were performed using the Illumina DRAGEN pipeline (v.4.0.3) using the official reference build GRCh38/hg38. GS included analyses for detection of both single nucleotide variants (SNVs) and copy number variants (CNVs), and the approach was broad without limitation to specific genes or a gene panel. To optimize the accuracy of results and maintain consistency in phenotypic descriptions, detailed fetal phenotypic data in the form of Human Phenotype Ontology (HPO) terms were utilized for variant prioritization [1, 2]. Variant interpretation was performed using Invitae Moon software (v4.0.2). A multidisciplinary genomics board reviewed the variant in the context of clinical history, and variant classification and confirmation were performed according to ACMG guidelines [3, 4].

## 3 Diagnostic Results and Interpretation

GS resulted at 30 weeks 5 days and revealed a heterozygous, de novo, likely pathogenic *FGFR2* variant (c.827 T > G, p.Phe276Cys) (Table 1B). Pathogenic *FGFR2* variants are associated with multiple craniosynostosis syndromes, including Antley‐Bixler, Apert, Crouzon, Saethre‐Chotzen, Pfeiffer, Jackson‐Weiss, and Beare‐Stevenson cutis gyrata syndrome (BSS) [5]. Ultrasound anomalies were thought to be most consistent with BSS.

## 4 Pregnancy Outcomes and Neonatal Findings

Given concern for airway compromise associated with BSS, delivery at our center was recommended [5, 6]. The patient presented in preterm labor at 35 weeks and delivered a male neonate (3.86 kg, > 97%) via repeat cesarean. The infant required significant intervention including immediate intubation and mechanical ventilation with assistance from Otolaryngology, which was difficult due to significant macroglossia and micrognathia. Clinical genetics evaluation noted brachycephaly, skin redundancy, proptosis, hypertelorism, natal teeth, cutis gyrata, edematous ears with linear divets, large/contracted left thumb, possible contractures of the digits, lower extremity 2‐3 syndactyly, right nipple skin tag, micropenis, hypospadias, large umbilical stump, facial hemangioma, and limited movement (Table 1A). The postnatal phenotype was felt to be consistent with BSS.

Plastic Surgery, Otolaryngology, Neurosurgery, Oral and Maxillofacial Surgery, and Ophthalmology evaluated the neonate and confirmed likely metopic craniosynostosis, possible choanal atresia, and ocular anomalies (Supplementary Material). Parents desired ongoing comfort care but no withdrawal of life‐sustaining technology due to faith‐based preferences. The infant expired on day of life 50.

## 5 Discussion

Specific gain‐of‐function *FGFR2* variants (p.Ser372Cys and p.Tyr375Cys) have been previously reported to cause BSS [5, 6]. This is the first report of a variant at this position (p.Phe276Cys) in association with BSS. Another substitution at the same position (p.Phe276Val) has been reported in unrelated individuals with craniosynostosis with clinical diagnoses such as Crouzon syndrome and *FGFR2*‐related craniosynostosis [7]. This highlights the value of detailed imaging to provide robust prenatal phenotyping and to facilitate appropriate counseling, anticipatory guidance, and multidisciplinary clinical care.

This case expands the genotype‐phenotype correlation of BSS and demonstrates the clinical utility of prenatal SNV analysis available via modalities such as GS, given that the genetic diagnosis had direct implications for delivery planning. The diagnostic evaluation emphasizes potential limitations of prenatal phenotyping, as concern for craniosynostosis did not arise until late in gestation, similar to BSS cases reported by Harada et al. [8] Lastly, our experience highlights the need for multidisciplinary counseling to support a family's faith‐based goals for care.

## Funding

The genome sequencing performed was funded by the NIH (R01HD107190 supporting Dr. Sparks). The contents of this publication are solely the responsibility of the authors and do not represent the official views of the funders. The authors received no funding for the preparation of this manuscript.

## Ethics Statement

The family reported in this manuscript signed informed consent to undergo genome sequencing through a clinical trial funded by the NIH. This trial received Institutional Review Board approval from the University of California, San Francisco.

## Consent

Genome sequencing was performed as a clinical service. The retrospective collection of data in this case is exempted from informed consent since all data presented have been anonymised.

## Conflicts of Interest

Dr. Sparks is an Associate Editor for Prenatal Diagnosis. The remainder of the authors on this study report no conflicts of interest.

## Supporting information


Supporting Information S1

## Data Availability

The data that support the findings of this study are available from the corresponding author upon reasonable request.
