# Supplementary material for: Prenatally Diagnosed Beare‐Stevenson Cutis Gyrata Syndrome With a Novel FGFR2 Variant
Source: Prenat Diagn. 2026 Mar 3;46(4):589–92. doi: 10.1002/pd.70113 (PMC13070219; doi:10.1002/pd.70113)
Supplement: Supplementary file 1 — Supporting Information S1 [file PD-46-589-s001.docx]

**Supplementary Materials:**

**Computed Tomography (CT) scans performed in our case:**

**CT Cervical Spine:** The C3, C4, C5 and C7 vertebral bodies are mildly hypoplastic. There are butterfly vertebral bodies of C2 base and C3. There is fusion of the left posterior elements of C2-C3 and C4-C5. There is fusion of the right posterior elements of C3-C4, C5-C6 and to a lesser degree C2-C3 (membranous).

**CT Head:** There is premature closure of the bilateral lambdoid sutures and partial premature closure of the sagittal suture. Partial closure of the metopic suture with ridging. Resultant brachycephalic and somewhat cloverleaf deformity of head with posterior flattening and osseous protuberance of the occipital tuberosity. There is partial permanent closure of the metopic suture with associated bridging. Remainder of calvarial sutures are patent. There is abnormal marked widening of the mastoid and sphenoid fontanelles with hypoplasia of the squamosal portions of the temporal bones. There are multiple convolutional markings throughout the calvarium. There is marked bilateral choanal atresia, likely with osseous and membranous components. There is atresia of the external auditory canals and hypoplasia of the middle ear cavities with suggestion of dysmorphic ossicles.
